# Study on the effect of light distribution on the greenhouse environment in Chinese solar greenhouse

**DOI:** 10.1371/journal.pone.0328302

**Published:** 2025-08-07

**Authors:** Weiwei Cheng, Yu Li, Liqiang Wang, Zhi Zhang, Zhonghua Liu

**Affiliations:** 1 College of Urban and Rural Construction, Shanxi Agricultural University, Jinzhong, China; 2 College of Agricultural Engineering, Shanxi Agricultural University, Jinzhong, China; Agricultural Sciences and Natural Resources University of Khuzestan, IRAN, ISLAMIC REPUBLIC OF

## Abstract

Solar greenhouse is a primary agricultural facility in northern China during winter, providing a certain level of security for the demand for vegetables and melons in the northern regions. However, there remains a lack of uniformity between crop requirements and the light and thermal environment within the planting area of the greenhouse, resulting in non-uniform growth and development of crops. The present study set out with the objective of investigating the impact of the light environment on the internal conditions of a solar greenhouse. To this end, experimental measurements were employed in conjunction with deep learning models. The results showed that rates of change in air temperature and light intensity were significantly higher in the vertical than the horizontal direction, especially below 1,800 metres, where significant differenced in temperature and light distribution existIn the horizontal direction, the impact of light distribution on soil temperature was significant within a range of less than 4,500 mm from the southern base of the greenhouse. By contrast, the impact was less pronounced within a range of 4,500 to 9,000 mm, In the temporal dimension, light variation significantly affected soil temperatures within 150 mm of the surface, but had no significant effect on temperatures within the 300–600 mm range. Similarly, light variation significantly affected temperatures within 200 mm of the inner wall surface, but had no significant effect on temperatures within the 400–800 mm range.Furthermore, vertical differences in light intensity significantly affected temperatures within the 800 mm height range from the indoor ground level, whereas the impact at other heights was less pronounced. The LSTM prediction model was highly accurate, and this study provided the necessary data and theoretical basis for regulating the light and temperature environments in solar greenhouse.

## 1. Introduction

With the changes in the world economy and climate, there is a big difference in vegetable production in winter between the southern and northern regions of China, resulting in expensive vegetables in the northern region [1]. The Solar greenhouse plays an indispensable role in winter crop cultivation in cold regions of China and is of strategic importance in ensuring year-round supply of agricultural products [2]. The solar greenhouse uses sunlight to power its operation and to promote crop growth. It uses walls, sheeting and quilts to create a microclimate suitable for growing crops [3].However, there is still the problem of unsuitable crop growth and environment in solar greenhouse.

Temperature and light in a solar greenhouse are pivotal environmental factors that impact crop growth [4], with solar radiation exerting a profound influence on the cultivation environment [5]. Sunlight penetrating the solar greenhouse membrane enables indoor crops to undergo photosynthesis, while the walls and floor of the greenhouse absorb solar radiation and store heat. This creates an ideal microclimate for plant growth during cold winter nights.

However, the regulatory measures of crop photosynthesis and the effects of light differences on the greenhouse thermal environment are not yet known, highlighting the importance of indoor solar radiation research. Consequently, conducting a comprehensive investigation into the impact of solar radiation on crop photosynthesis and the thermal environment within a greenhouse is imperative for the optimisation of greenhouse design and the enhancement of crop yields.

To maximise the capture of solar radiation and increase indoor temperature at night in winter, researchers have focused on the absorption and transmission of solar energy by thin film [6]. However, there was no significant difference in the total amount of solar radiation entering the interior of the greenhouse through the different curvatures of the south roof [7], and the effect of the difference in indoor light on the thermal environment of the greenhouse remains unknown [8]. Therefore, it is equally important to study the indoor solar radiation differences and the weighting of the indoor thermal environment effects they cause [9].

Many scholars have studied the radiation model for solar greenhouses [10]. In a solar greenhouse, solar energy is the most important and economical source of heat.

To increase the amount of solar radiation entering the solar greenhouse, many studies have focused on optimising the design parameters of its southern side. Research has been conducted to calculate and analyse the solar radiation received by greenhouses of different dimensions in southern China. The aim was to optimise the design of solar greenhouses according to the characteristics of different latitudes [11]. Utilising the principles of solar motion, meteorological data and the optical properties of materials, researchers have developed a novel solar radiation model. They have further explored the effect of ridge height on the solar radiation capture efficiency of a greenhouse and proposed a design to optimise the ridge height [12]. In the study of roof form, researchers analysed the effect of different roof designs on the amount of indoor solar radiation using a two-dimensional solar radiation calculation model. This model demonstrated that the front roof form and the angle of inclination of the roof play a crucial role in enhancing the level of solar radiation in a greenhouse [13]. Conversely, studies on roof curvature have demonstrated that the impact of south-facing roofs with varying curvatures on the quantity of solar radiation in a greenhouse was not substantially different [14]. These results provided important theoretical support for optimising the design of solar greenhouses and improving their capacity to capture solar radiation.

In order to explore the amount of solar radiation entering solar greenhouse, researchers employed mathematical models in order to calculate the solar greenhouse aspect ratios for six cities of varying altitudes. The aspect ratios were then compared in order to highlight the differences in solar radiation between sunny and snowy conditions. Finally, the effects of four different aspect ratios on the equivalent transmittance of beam radiation, scattered radiation and ground reflected radiation under the same sunny conditions were analysed. The results of the study showed that the intensity of reflected radiation from sunlight was high at high altitudes [15]. Utilising meteorological data and the established laws of motion of the Earth and the Sun, the researcher proceeded to investigate the variation of solar rays and the incidence angle of the front roof surface of the solar greenhouse. The researcher then established a model of solar radiation in the greenhouse and verified it by experimental data. This showed that the radiant irradiation of the ground was higher than that of the wall surface during the equinoxes to the autumn equinoxes, while the solar radiant irradiation of the wall surface was greater than that of the ground during the autumn equinoxes to the spring equinoxes [16]. Furthermore, Zhang [14] studied the distribution of solar radiation in solar greenhouse using a two-dimensional solar radiation calculation model. This model demonstrated that a solar greenhouse is suitable for crops requiring 42,000–57,200 lx of illumination in spring and summer, and 25,000–40,000 lx of illumination in autumn and winter. In an academic study, researchers constructed a solar radiation model for solar greenhouse that included the effect of shading. This study introduced the concept of utilisation efficiency in order to explain the differences in indoor radiation distribution. During the winter months, a shift in the peak surface solar energy was observed, with a transition from the south-west to the south-east. Furthermore, the results demonstrated that the radiation reception at the corner locations was significantly lower [9]. Finally, cheng [17] analysed the rate of light change in a solar greenhouse based on a mathematical model, and the results showed that light change was most intense along the vertical direction, followed by the horizontal direction, with the least change in the vertical direction. A comprehensive array of temperature measurement points were meticulously configured within the greenhouse to validate the model. The study revealed discrepancies between the measured and theoretical light distributions, yet these findings provided substantial insights and reliable data on the complex relationship between light distribution and crop growth in actual greenhouse environments.

In order to gain an in-depth understanding of the distribution of light environment in solar greenhouse, many scholars have conducted extensive experimental studies. El-Maghlany [18] conducted a study into the effect of different positional orientations and elliptical aspect ratios on solar radiation. The incoming solar radiation was calculated by means of a mathematical model, which demonstrated that the capture of solar radiation was closely related to the ratio between the area covered by a greenhouse and the area of cultivated land. Wang [19] conducted a study to measure the light environment in the greenhouse. Combining this data with total solar radiation data from external sources revealed a consistent higher light intensity of solar radiation on the south side of the greenhouse compared to the north side. However, due to the limited data available, it was not possible to derive a clear pattern. Liu [15] constructed a solar radiation model for the ground and wall of a solar greenhouse. The model explored the effects of different seasons, roof forms and front roof inclination on the total solar radiation. It also developed a calculation method for the distribution of diffuse solar radiation. This provided a theoretical basis for the further analysis of the light environment in the greenhouse. Chen [20] established that the heat storage and exothermic effect of the mountain wall significantly influenced the indoor temperature and radiation distribution. This finding was derived from continuous observations of the heat flux in a solar greenhouse. The study indicated that a higher shading rate resulting from the hill wall in short-length a greenhouse led to a reduction in solar radiation received. It was proposed that enhancing the length of the greenhouse, optimising the angle of the hill wall, and rationally planning the crop layout could lead to improvements in light utilisation and overall thermal efficiency. In the study of the shading effect of the mountain wall, Han [21] established an estimation model of the shadable view angle of the mountain wall and simulated it through the use of the VB programming language. The model predicted the spatial distribution of solar radiation in the greenhouse and was verified by comparison with measured data. The study estimated the difference in the distribution of beam radiation and diffuse radiation, thereby revealing the influence of the enclosure structure on the light environment in the greenhouse. In a study by Yang [22], the impact of roof inclination on the light environment of a solar greenhouse was investigated through a simulation-based approach. Key indicators such as light transmittance, radiant intensity and cumulative light radiant energy were analysed. The results showed that with an inclination of 32°, a significant enhancement in the cumulative light and a substantial improvement in the light environment of the back roof were observed. These findings indicated that an increased roof inclination could promote optimal growth conditions for crops. The above studies have provided a large amount of data and theoretical support for the optimisation of the greenhouse light environment, but most of these experiments have focused on the effects of single factors and lacked a comprehensive analysis of the distribution of the light environment across the entire greenhouse cross-section. Therefore, they have not yet provided sufficient basis for the rational cultivation of sun-loving crops.

In order to meet the needs of the majority of sun-loving crops cultivated in a greenhouse, researchers have carried out a number of experiments with the aim of enhancing solar energy utilisation in a greenhouse. Zhen et al. [23] measured the change in quantum yield of lettuce in response to the addition of narrow-band far-red light. The study revealed that photons with wavelengths ranging from 700 to 732 nm significantly enhanced photochemical efficiency. However, photons with wavelengths exceeding 752 nm did not demonstrate such an effect. Masakazu Nakayama [24] and others conducted a study in which they supplemented LED lighting for greenhouse-grown strawberry of different varieties. The study demonstrated that the photosynthetic quantum flux density (PPFD) of the crop canopy averaged 237.0 $\mu mol\cdot m^{-2}\cdot s^{-1}$ at night. This value was then compared to that of a conventional greenhouse, which resulted in an increase in the cumulative PPFD during the growing season by 951.0 $\mu mol\cdot m^{-2}\cdot s^{-1}$. However, this increase was accompanied by an increase in energy consumption. In their seminal paper, Shi et al. [25] proposed a novel methodology for optimising the light environment through the use of reflective films. This methodology resulted in a 5.33% increase in total solar radiation on the inner surface of the CSG. However, it was also observed that when the inclination angle of the north wall was set between 15° and 25°, the light interception rate was reduced by 7.91% to 10.54%. While the position and angle of installation of the reflective film had minimal impact on the crop canopy, the benefit of additional light was insufficient to compensate for its shading effect. In order to enhance the efficiency of full-spectrum utilisation within a greenhouse, Ma [26] et al. combined spectral splitting technology with agricultural production in order to design a novel greenhouse cover structure. This structure was capable of separating and utilising visible (VIS) and near-infrared (NIR) light, thus significantly improving the efficiency of integrated full-spectrum solar energy utilisation in a greenhouse. In addressing the limitations of conventional light replenishment systems employed in facility agriculture, Cheng and colleagues [27] have devised an intelligent light replenishment system founded on the particle swarm optimisation-support vector regression (PSO-SVR) algorithm. This system employed greenhouse environmental information to predict the light saturation point, thereby enhancing operational efficiency and sustainability. The experimental results demonstrated the system’s stability and the ability to reduce energy consumption by approximately 10%, effectively promoting intelligent regulation of the light environment and facilitating energy savings and emission reduction. However, it should be noted that, in terms of hardware, the memory and CPU occupancy of the system were high, which has a detrimental effect on the performance of the system. Despite this, however, further research is required to improve the understanding of the relationship between plant photosynthesis and light radiation, with the aim of facilitating more precise regulation of the environment in which crops grow.NIU [28] et al. utilized a genetic algorithm to optimize the supercritical CO₂ Brayton cycle system, significantly enhancing its thermal efficiency and overall performance. This study demonstrated the value of optimization algorithms in complex systems. Similarly, in the control of solar greenhouse, optimization methods combined with deep learning algorithms can be applied to dynamically model the greenhouse environment. By collecting and analyzing extensive experimental data, these methods can accurately predict the greenhouse environment [29].

The continuous development of deep learning technology has prompted numerous scholars to explore the application of deep learning prediction models to optimise the growing environment of crops. Wang [30] et al. selected a recurrent neural network (RNN) model as a deep learning algorithm in the domain of climate prediction and employed a dynamic Bayesian optimisation (BP) method to recalibrate the model parameters, a combination of which exhibited superior performance in temperature and humidity forecasting. Cheng et al. [31] established an internal environment simulation model based on meteorological observation data in the solar greenhouse, combined with an Elman neural network. Through simulation and verification of time-by-time and day-by-day, the Elman neural network was found to outperform the traditional BP neural network in terms of accuracy and stability. Concurrently, Taewon Moon et al. [32] sought to enhance a BiLSTM model via transfer learning techniques for the prediction of environment variables, such as light, thereby demonstrating the efficacy of transfer learning in enhancing model adaptability and accuracy in contexts where agricultural data was limited. Liu [33] et al. proposed a GCP-LSTM greenhouse climate prediction model, which was based on LSTM to capture the nonlinear dependence between historical climates and effectively control the greenhouse climate to ensure stable crop growth. In a related study, Yang [34] et al. constructed an environmental prediction model integrating gradient boosting tree and Harris hawk optimisation algorithm (IHHO-Catboost). This model comprehensively considered the internal and external environmental factors and regulatory factors affecting crop growth. These factors were incorporated into a prediction model with time-series characteristics, which had a higher prediction accuracy compared to the LSTM model and BP neural network. In a seminal work, Zu [35] et al. presented an LSTM greenhouse environment prediction model based on SSA optimisation. This model demonstrated a remarkable capacity to accurately predict a broad spectrum of environmental variables, including greenhouse light. Notably, it exhibited higher accuracy in this regard compared to competing models such as BP, GRU, and LSTM. Additionally, Zang [36] proposed a deep learning spatio-temporal correlation model combining a convolutional neural network (CNN) and a long short-term memory network (LSTM). The results of the study demonstrated that the model was capable of accurately predicting global horizontal irradiance one hour in advance. These studies have enhanced the precision of environmental prediction within the temperature chamber, thereby facilitating more intelligent and efficient crop growth regulation [37]. However, there is still a necessity to further refine the model to optimise the precise regulation of the crop growth environment.

Furthermore, considering the impact of light saturation points on light utilization, Niu et al. [38] constructed a photosynthesis prediction model using the least squares support vector regression, enhancing the accurate forecasting of Pn under complex lighting conditions, that was, when spectral and light intensity were coupled. This improved crop photosynthesis while reducing carbon emissions from SL by 14.85%, which was of significant importance for carbon reduction and energy saving in greenhouse light environment regulation and promotes the application of multi-spectral regulation in greenhouse environments. R et al. [39] utilized deep learning models to process time series data for predicting solar irradiance and photovoltaic (PV) power. Three separate models and one hybrid model were discussed. Most studies indicated that the hybrid model outperformed the three individual models in forecasting solar irradiance, demonstrating that deep learning models were better suited for predicting solar irradiance and PV power. Xin et al. [40] successfully determined the light saturation point for cucumber photosynthesis by combining multi-factor experiments with an SVM photosynthetic rate prediction model, laying a theoretical foundation for the optimization and control of the greenhouse light environment.

As was apparent from the existing body of literature, considerable progress has been made in relation to the establishment of regulatory mechanisms for the light environment within solar greenhouse. Notwithstanding, there remains certain deficiencies in the quantitative analysis of light within the various regions of the greenhouse, the extent of which precluded the realisation of precise regulation of the light environment. The majority of extant research concentrated on the description of the overall light environment, but lacked a detailed quantitative assessment of the light level in each region inside the greenhouse. This results in the light environment regulation not being spatially accurate. Furthermore, the microenvironmental climate in the greenhouse is closely dependent on changes in the light environment. However, existing studies have failed to provide long-term, multi-point, continuous measurements of the light environment. This limitation hinders the precise numerical characterisation of the dynamics of the light environment, consequently affecting the accurate response of the regulation system to the greenhouse climate. Consequently, extant greenhouse control technology has thus far been incapable of effecting effective targeted adjustment, nor of demonstrating sufficient flexibility in order to satisfy the light requirements of specific areas, with the result that the optimisation of the crop growth environment has been compromised.

To address the current insufficiencies in the quantitative analysis of light in different greenhouse areas, we deployed measurement points within the greenhouse and conducted long-term continuous measurements. Through experimental studies and deep learning models, we investigated the light environment in various greenhouse regions. This approach clarified the spatial distribution of the light environment and the boundaries between different light intensities, thereby providing data support for precise regulation.

## 2. Materals and methods

### 2.1 Experimental greenhouse

**Fig 1:**
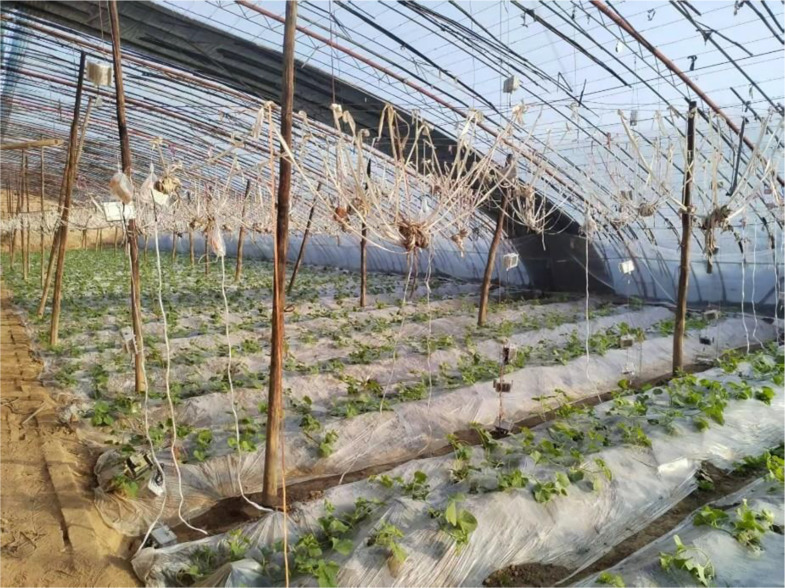
Solar greenhouse.

As illustrated in Fig 1, the experimental greenhouse has been situated in the Taigu, Jinzhong, Shanxi, China. The facility under investigation was a conventional passive solar greenhouse, which sustained the greenhouse environment by accumulating solar energy through heat storage media and subsequent exothermic heat. The dimensions of the structure were as follows: its length was 75 m in the north-south direction, its width was 10 m, and its ridge height was 4.0 m. The east-west hill wall has a span of 3 m on the south side, and the north wall was a trapezoidal earth-built wall with a width of 8 m at the bottom and 2 m at the top. The top was erected with about 2 m long wooden sticks to the skeleton and covered with PO film. The primary structure of the greenhouse comprises 28 elliptical steel tubes, arranged at intervals of 2.8 metres, with the western exitway providing access to the preparation room.

The test greenhouse was planted with melons on 11 December 2024. The planting method involved the use of raised bed ground cover in combination with drip irrigation. An 1 m wide pedestrian aisle was situated on the north side, with rows spaced approximately 450 mm apart and a spacing of 100 mm between the crops. The outermost layer of the planting area was situated 1,000 mm from the south bottom corner.

### 2.2 Test materials

**Fig 2:**
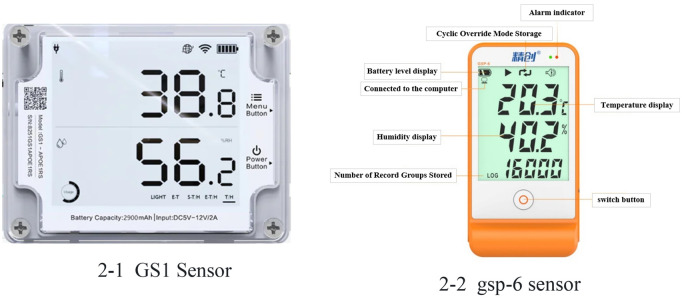
Test equipment.

Air temperature, humidity and light: This test used an easy-to-connect GS1 industrial temperature and humidity recorder to measure indoor light intensity and carbon dioxide concentration, with a total of 42 sensors deployed. Its temperature range was −20°C-60°C with an accuracy of ±0.3°C; humidity range was 10%−90% with an accuracy of ±3% RH; light range was 0.01-157k lux with an accuracy of ±2%, and CO_2_ measurement range was: 0 ~ 40000 ppm, as shown in Fig 2-1.

Temperature and humidity of soil and wall: The temperature and humidity measuring instrument for soil and wall were selected from Varitronix GSP-6, which complied with CE (EN12830), RoHS and FDA (21CFR) standards, and the data was recorded every 10 min, with a total of 34 sensors deployed. The measurable temperature range of the device was −40°C ~ 85°C, and the temperature accuracy was ± 0.5°C in the temperature range (−20°C, + 40°C), and ±1.0°C in the other measurable temperature ranges, as shown in Fig 2-2.

### 2.3 Test method

According to the group’s previous research [41], the magnitude of variation of the thermal environment of the greenhouse was greater along the vertical and horizontal directions, and the central cross section was selected at the central position of the greenhouse in the east-west direction, avoiding the space covered with quilts in the middle, and the test period was from 24 December 2024 to 29 February 2025.

**Fig 3:**
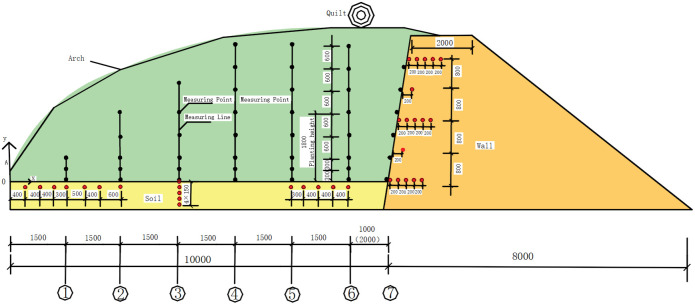
Layout of measurement points and lines.

Location of air measurement points: In the central cross-section of the greenhouse, seven positions were selected along the north-south direction at intervals of 1500 mm. Pulleys were installed at these positions to suspend temperature sensors at different heights. Three measurement points near the ground were spaced at 300 mm intervals, while the remaining points were spaced at 600 mm intervals. This configuration resulted in a total of seven measurement lines with 42 sensors. A coordinate system was established with the southern base of the cross section ridge in the centre of the span as the origin, vertically upwards as the y-axis and northwards as the x-axis. Line 1 was at the southern start of the melon planting area, line 4 was in the middle of the north-south direction, line 6 was at the northern end of the melon planting area and line 7 was on the surface of the northern wall. The coordinates of the air measurement points were shown in Fig 3 and as shown in Table 1.

**Table 1:** Location of measurement points in the greenhouse air.

Measuring Line number								
		1	2	3	4	5	6	7
Measuring point number	*X*/m	1500	3000	4500	6000	7500	9000	10000
	1	0	0	0	0	0	0	0
	2	0.3	0.3	0.3	0.3	–	0.3	–
	3	0.6 (CO_2_)	0.6 (CO_2_)	0.6 (CO_2_)	0.6 (CO_2_)	0.6 (CO_2_)	0.6 (CO_2_)	0.6
	4		1.2	1.2	1.2	1.2	1.2	1.2
	5		1.8	1.8	1.8	1.8	1.8	1.8
	6			2.4	2.4	2.4	2.4	2.4
	7				3	3	3	3
	8					3.6	3.6	

Layout of the soil points: Seven measurement points were arranged at y = −150 mm, within the range of x=(400,3000)mm, the measurement points were spaced at intervals of 400 mm, 400 mm, 300 mm, 500 mm, 400 mm, and 600 mm, extending from south to north; In the range of x=(7500,9000)mm, five measurement points were arranged with intervals of 300 mm, 400 mm, 400 mm, and 400 mm from south to north. At x = 4500 mm, five measurement points were arranged at y = 0 mm, −150 mm, −300 mm, −450 mm, and −600 mm. A total of 17 soil measurement points were deployed, with their coordinates as shown in Fig 3 and Table 2.

**Table 2:** The layout of soil temperature measurement points in daylight greenhouse (unit: mm).

coordinates	Measurement point number												
	S1	S 2	S 3	S 4	S 5	S 6	S 7	S 8	S9	S 10	S 11	S 12	S 13
X	400	800	1200	1500	2000	2400	3000	4500	7500	7800	8200	8600	9000
Y	−150	−150	−150	−150	−150	−150	−150	0	−150	−150	−150	−150	−150
								−150					
								−300					
								−450					
								−600					

Location of wall measurement points: Temperature and humidity measurement points were located at heights of y = 0 mm, 1600 mm and 3200 mm, and the horizontal distance from the inner surface of the north wall was 0 mm, 200 mm, 400 mm, 600 mm and 800 mm. Arrangement of temperature and humidity measuring points at heights of y = 0 mm, 800 mm, 1600 mm, 2400 mm and 3200 mm and at a horizontal distance of 200 mm from the inner surface of the wall. The coordinates of the north wall measurement point arrangement were as shown in Fig 3 and as shown in Table 3.

**Table 3:** Layout of wall measurement points (unit: mm).

section	Height of wall cross section	Measurement point number				
		1	2	3	4	5
W1	3200	0	200	400	600	800
W2	2400	–	200	–	–	–
W3	1600	0	200	400	600	800
W4	800	–	200	–	–	–
W5	0	0	200	400	600	800

### 2.4 Classification of measuring points

#### 2.4.1 Light-weighting method.

The light data of each measurement line was weighted in order to create a trend of light change over time at different locations. This was followed by a preliminary judgement on the classification of light intensity. The weighted light formula of the measurement line was as follows:


$$L_{x}=\frac{\sum_{i=1}^{n}l_{xi}y_{i}}{\sum_{i=1}^{n}y_{i}}$$

The location of the line is indicated by *L*, the light value of the line, while the point is denoted by *l*, the light value of the point. The x-coordinate of the line was expressed as *x*, and the y-coordinate of the point as y. Finally, *n* signified the number of points in the line.

#### 2.4.2 k-means classification.

In order to ascertain the optimal number of classifications for K-Means clustering, the Elbow Method can be utilised. The method ascertained the optimal number of clusters by observing the rate at which the variation within clusters diminished. In particular, it computed the total sum-of-squares error (SSE) for differing numbers of clusters and sought an ‘elbow point’, that is to say, a point at which the SSE diminished significantly less as the number of clusters increased.

K-Means clustering is a classical algorithm in unsupervised learning for dividing data points into a predetermined number of clusters, making the data points within each cluster relatively more similar. The algorithm commences by selecting K points at random as the centre of mass. It then proceeds to classify the data points into the nearest clusters based on their Euclidean distance to the centre of mass. Subsequently, the recalculation of the centre of mass of each cluster is undertaken by the algorithm, which then re-classifies the data points based on the new centre of mass. This process is iterated until either the change in the centre of mass is less than a specified limit or a predetermined number of iterations is reached, thus ensuring maximal homogeneity within the clusters.

Employing the K-means algorithm, we classified the weighted light values for different measurement lines during distinct time slots. Drawing on the light classification by Wang Junheng [30], we divided the light intensity at various times and locations within the greenhouse into weak ($\leq 1.5\times 10^{4}lx$), moderate ($1.5\times 10^{4}lx,2.5\times 10^{4}lx$), and strong ($\geq 2.5\times 10^{4}lx$) categories. This approach allows for an analysis of temperature and light during different time periods, identifying the temperature differences resulting from light variations at different measurement points.

### 2.5 LSTM prediction

Long Short-Term Memory (LSTM) is a state-of-the-art recurrent neural network architecture specifically designed to solve the long-term dependency problem encountered by traditional RNNs when processing sequence data.LSTM significantly improves the model’s ability to capture long-term dependency information in sequences by introducing three key gating mechanisms: an input gate, a forgetting gate and output gates, as well as a cell state.

#### 2.5.1 The basic unit structure of LSTM.

In the LSTM architecture, the computation at each time step *t* is accomplished through the coordinated efforts of the forget gate, input gate, cell state, and output gate. The forget gate employs a Sigmoid function, with values ranging from [0, 1], to determine which information to discard from the cell state. The input gate decides which new information to store, with its activation values computed by a Sigmoid function and the candidate cell states computed by a Tanh function, whose values range from [−1, 1]. The cell state is updated by multiplying the forget gate output with the previous state and adding the product of the input gate activation and the candidate state. The output gate uses a Sigmoid function to decide which cell state information to output. The final hidden state *ht* serves as the unit output, which is passed to the next time step. This mechanism effectively captures long-term dependencies in sequences.

The computation of the LSTM model at time step t is described in detail as follows, where the input is (*x*_1_,*x*_2_,...,*x*_t_), the hidden layer output is (*h*_1_,*h*_2_,...,*h*_t_), and the cell state is (*C*_1_,*C*_2_,...,*C*_t_):


$$\begin{array}{c} f_{1}=\sigma \left(W_{f}\cdot \left[h_{t-1},x_{t}\right]+b_{f}\right)\\ \;\;\;\;\;i_{t}=\sigma \left(W_{i}\cdot \left[h_{t-1},x_{t}\right]+b_{i}\right)\\ {\tilde{C}}_{t}=\tanh\left(W_{C}\cdot \left[h_{t-1},x_{t}\right]+b_{C}\right)\\ \;\;\;\;C_{t}=f_{t}\cdot C_{t-1}+i_{t}\cdot {\tilde{C}}_{t}\\ o_{t}=\sigma \left(W_{o}\cdot \left[h_{t-1},x_{t}\right]+b_{o}\right)\\ \;\;\;\;h_{t}=o_{t}\cdot \tanh(C_{t}) \end{array}$$

where $f_{t}$ represents the output of the forgetting gate, which determines what information needs to beforgotten from the cell state; $i_{t}$ is the output of the input gate, which decides which new information will be added to the cell state; $\overset{\sim }{C_{t}}$ denotes the candidate cell state, i.e., the candidate for new information; $C_{t}$ is the cell state at the current time step; $O_{t}$ is the output of the output gate, which decides which information in the cell state will be outputted; and $h_{t}$ is the output of the hidden layer at the current time step, and also the output of the LSTM cell. The weight matrices for each gate are$W_{f}$,$W_{i}$,$W_{c}$ and$W_{o}$, and the corresponding bias terms are$b_{f}$,$b_{i}$,$b_{c}$, and$b_{o}$, respectively $h_{t-1}$ is the hidden state of the previous time step, $x_{t}$ is the input of the current time step, and $C_{t-1}$ is the cell state of the previous time step.

#### 2.5.2 The hierarchical structure of the LSTM model.

In practical applications, LSTM models typically consist of multiple layers of LSTM units, which enhance the model’s ability to capture complex temporal dependencies. The LSTM model constructed in this study is a Bidirectional Long Short-Term Memory Network (Bidirectional LSTM). This model is designed to enhance the capture of long-term temporal dependencies in the time series data. The model consists of two layers of LSTM units. The first layer is configured with 256 neurons and returns the full sequence by setting “return_sequences=True”, ensuring that the next layer receives the complete temporal information. The second LSTM layer, which immediately follows, contains 128 neurons and returns only the final output of the sequence by setting “return_sequences=False” for subsequent processing steps. To mitigate the over fitting phenomenon, a Dropout layer was added after each LSTM layer, and the Dropout ratio was set to 0.3. This effectively reduces the risk of over fitting to the training data. Finally, the output layer of the model is a Dense layer with 2 neurons, corresponding to the predicted values of temperature and light. A linear activation function is selected for the regression task to ensure accurate prediction results.

#### 2.5.3 Data preprocessing.

The pre-processing of the experimental data comprised the treatment of outliers and missing values, data standardisation, and for both missing values and outliers, the average of the two moments before and after the value was selected to replace the value at that moment. The standardisation of the data was undertaken to enhance the accuracy of model training and expedite the convergence rate. The formulas used for handling missing values, outliers, and data normalization are as follows:


$$X_{filled}=\frac{X_{t-1}+X_{t+1}}{2}$$

$$X_{par}=\frac{X-X_{\min}}{X_{\max}-X_{\min}}$$

Where $X_{filled}$ denotes the data after the treatment of missing values and outliers; $X_{t-1}$ and $X_{t+1}$ represent the data from the two time points immediately before and after the moment of interest, respectively; $X_{par}$ is the data after standardisation; *X* represents the original data; $X_{\max}$ is the maximum value in the original dataset; $X_{\min}$ is the minimum value in the original dataset.

#### 2.5.4 Model evaluation metrics.

The mean absolute error (MAE), mean square error (MSE), and coefficient of determination (R^2^) were used as model evaluation indexes.


$$\begin{array}{c} \text{MAE}=\frac{1}{n}\sum_{i=1}^{n}\left|\left(y_{i}-{\tilde{y}}_{i}\right)\right|\\ MSE=\frac{1}{n}\sum_{i=1}^{n}{\left(y_{i}-{\tilde{y}}_{i}\right)}^{2}\\ R^{2}=1-\frac{\sum_{i=1}^{n}{\left({\tilde{y}}_{i}-y_{i}\right)}^{2}}{\sum_{i=1}^{n}{\left({\bar{y}}_{i}-{\tilde{y}}_{i}\right)}^{2}} \end{array}$$

where *y*_i_ is the experimental data, ${\tilde{y}}_{i}$ is the predicted value and ${\bar{y}}_{i}$ is the mean value.

#### 2.5.5 Hyperparameter tuning.

To optimize the performance of the LSTM model, adjustments were made to the following hyperparameters:

(1) Learning Rate: The learning rate determines the step size at which the optimizer updates the model parameters. In this study, the default learning rate of the Adam optimizer (0.001) was used.
(2) Sequence Length: The sequence length (look_back) indicates the number of past time steps the model uses to predict the current time step’s value. In this study, look_back was set to 48, meaning that data from the past 48 time steps are used for prediction.
(3) Batch Size: The batch size refers to the number of samples used to update the model parameters in each iteration. In this study, the batch size was set to 64.
(4) Dropout Rate: The Dropout rate is utilized to prevent the model from overfitting. In this study, the Dropout rate was set to 0.3.

## 3. Results and analyse

The data of the lowest day of outdoor temperature in the period December 2024-February 2025 were selected for analysis to study the pattern of light distribution on the indoor thermal environment in the solar greenhouse. Based on the outdoor temperature data, the data of light, temperature and humidity, and CO_2_ at all measuring points on 29 January 2025 were selected for analysis.

When making light measurements in a solar greenhouse, the equipment may be subject to rotation and arc shading and the appropriate margin of error should be selected. Errors in data collection: equipment accuracy error, the accuracy of the equipment is ± 2%, which is the inherent error of the equipment itself; the rotation of the equipment may cause changes in the measurement angle, affecting the measurement of light intensity. According to a related study [42], when the angle of incidence exceeded 40°, it leaded to a large discrepancy between the calculated and measured values; arch shading reduces some of the light and affects the measurement results. Combining the above factors, a more conservative error range of 10% was used [43]. This error range can cover the effects of equipment accuracy, equipment rotation and bow shading to ensure the reliability of the measurement results.

### 3.1 Analysis of the effect of light on air temperature

On 29 January 2025, it was a sunny day with an outdoor minimum temperature of −16.3°C and a maximum temperature of 14.3°C. The daylight greenhouse had the quilts stowed at 8:45, the upper air vents were turned on at 12:11, and the upper air vents were closed and the quilts were lowered at 17:14. The data collected between 9:00 and 17:00 were selected for analysis.

By applying the K-Means clustering analysis method, the light intensity data from different measurement locations in the greenhouse at various times were clustered to determine the periods of weak lighting ($\leq 1.5\times 10^{4}lx$), moderate lighting ($1.5\times 10^{4}lx,2.5\times 10^{4}lx$), and strong lighting ($\geq 2.5\times 10^{4}lx$) throughout the day.

#### 3.1.1 Patterns of change in light and temperature along the vertical.

The measuring points at Y = 0 mm for measuring lines 1–6 were at the same height as the ridge and furrow, and the crop was planted with a ridge height of 300 mm, i.e., at the same height as the crop root. Measuring line No. 1 was at a horizontal distance of 1500 mm from the southern footing and 700 mm from the southern start of the crop, with four crops planted within this 700 mm area; measuring line No. 6 was at the termination of the crop and measuring line No. 7 was at the surface of the wall.

**Fig 4:**
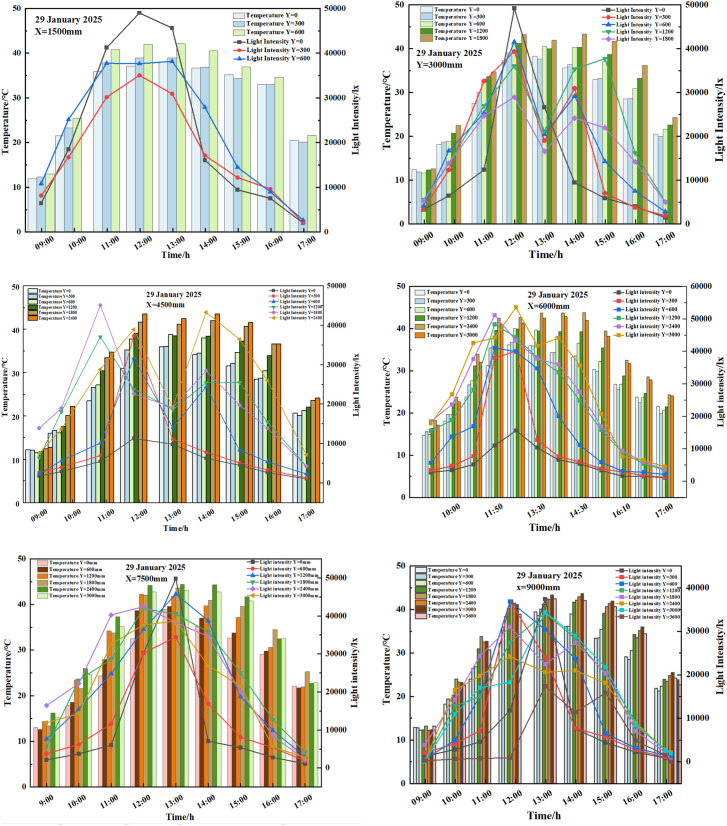
29 January 2025 Plot of light temperature variations at different heights over the same span.

As shown in Fig 4: the temperature in the solar greenhouse after opening the quilts, the indoor light intensity gradually increased, the temperature of all measurement points gradually increased, the maximum temperature difference between different heights of the same span of the greenhouse gradually increased, the greenhouse to reach the maximum temperature of the time concentrated in the 12:20 ~ 13:20 between the time, after which the temperature gradually decreased; The period of weak light intensity 1 in the morning was between 9:20 and 10:00 at each measurement point on lines 1–6 in the solar greenhouse, and the period of weak light intensity 2 in the afternoon was between 15:30 and 17:00; The medium light intensity 1 period of the morning time period was between 10:00 and 10:40, and the medium light intensity 2 period of the afternoon time period was between 14:30 and 15:30 at each measurement point of measurement lines 1–6 in the solar greenhouse; The strong light intensity in the morning hours was between 10:40 and 14:30 at each of the measuring points on lines 1–6 in the solar greenhouse; Due to the shading effect of the top arch on the top measuring point of each line, and the fact that line 6 was located at the north end of the crop planting, this position was located in the shadow range of the cotton cover, so the maximum light measuring point of the measuring line numbered 1, 2, 3, 4, 5, and 6 and the data of measuring point numbered 1 in the corresponding line in this time period were selected for analysis.

**Vertical patterns of temperature and light change during weak light hours.** During the period from 9:00–10:00 in the morning, which belonged to weak light zone 1, the temperature at the top measuring point before opening the greenhouse film was the lowest and the temperature at the No. 1 measuring point of each measuring line was the highest; After opening the greenhouse film, the light intensity of the measuring points in the higher part of the same span was greater and the temperature values increased faster, with an average outside temperature of 3.2°C and an average outside light intensity of 68,344.7 lx in the period of the weak light zone; The average temperature values of the measuring points at the top of the same line in measuring lines 1−6 were 18.9°C, 18.4°C, 19.8°C, 17.3°C, 19.7°C and 17.5°C, respectively, and the temperature difference between them and measuring point No. 1 of the corresponding line were 1.0°C, 0.2°C, 0.6°C, 0°C, 2.3°C and 0.3°C, respectively. Due to the shading of the top measuring point by the top arch, resulting in the shading of the light intensity at the top measuring point, the average temperatures of the maximum light measuring points 1−3, 2−5, 3−5, 4−7, 5−6, 6−5 in the measuring lines 1–6 were: 19.0°C, 18.4°C, 18.3°C, 19.9°C, 20.9°C, 18.1°C; and the average temperature difference with the measurement point No.1 of the measurement lines were 2.7°C, 2.7°C, 3.2°C, 3.5°C, 5.6°C, 2.7°C, respectively; the average light intensity at the maximum light measurement points 1−3, 2−5, 3−5, 4−6, 5−6, 6−5 in the measurement lines 1 ~ 6 were 14562.6 lx, 10843.8 lx, 19547.1 lx, 19632.7 lx, 20740.1 lx and 9329.3 lx. The difference in mean light intensity at the corresponding measurement point No.1 were 2156.9 lx, 2156.8 lx, 5832.4 lx, 16613 lx, 17842.4 lx, 7199 lx.

In the afternoon time period from 15:30–17:00, it belonged to the low-light zone 2, and the temperature of the measuring point No. 1 of each measuring line was the highest in this time period; the light intensity of the measuring point at the higher part of the same span height was larger, and the temperature of the measuring point of each measuring line gradually showed a decreasing trend with time; the average outdoor temperature in the low-light zone time period was −1.2°C; The average temperature values of the measuring points at the top of the same measuring line in measuring lines 1−6 were 26.3°C, 30.8°C, 31.1°C, 29.2°C, 29.8°C, 30.8°C, respectively, and the temperature differences between them and the corresponding measuring point No. 1 of the measuring line were −3.7°C, 5.8°C, 6°C, 4.5°C, 3.3°C, 4.9°C, respectively. Due to the shading of the top arch to the top measuring point, resulting in the shading of the light intensity of the top measuring point, the higher temperature of measuring point 1 in line 1 is 30°C, and the light intensity was 6861.9 lx;, the average temperatures of the maximum light measuring points 2−5, 3−6, 4−7, 5−5, 6−6 in lines 2 ~ 6 were: 30.9°C, 31.1°C, 29.2°C, 31.6°C, 30.8°C; and the average temperature difference between the measuring point 1 in each line were 6°C, 6°C, 4.4°C, 5.4°C, 4.9°C, respectively. The average temperature difference of measuring point No.1 of each measuring line were 6°C, 6°C, 4.4°C, 5.1°C, 4.9°C; the average light intensity of the largest light measuring points 2−5, 3−6, 4−7, 5−5, 6−6 in the measuring lines from 2 to 6 were: 10843.8 lx, 14918 lx, 7531.2 lx, 9402.2 lx, 6541.8 lx, and the average light intensity difference of measuring points corresponding to the measuring point No.1 were: 10843.8 lx, 14918 lx, 7531.2 lx, 9402.2 lx, 6541.8 lx. The difference in mean light intensity at the corresponding point 1 were:2156.8 lx, 5832.4 lx, 5806.7 lx, 6793.7 lx, 4822.1 lx, respectively.

Based on the above analysis, it can be seen that: in the weak light period, removing the influence of the arch and the blanket at the upper measuring point of each measuring line No. 1 and No. 6, the light intensity at the higher measuring point of the same span from the ground was greater, and the temperature value of the top measuring point was higher; As the light intensity of the top measurement point of the same measuring line was greater, its temperature value was higher; the temperature of the weak light 1 period was between 17°C ~ 20°C, and the maximum light was between 9000 lx ~ 21000 lx; the temperature of the weak light 2 period was between 24°C ~ 32°C, and the maximum light was between 6000 lx ~ 15000 lx; For the same line, the light difference between the lightest point and its counterpart, point 1, was greater in the morning than in the afternoon, but the temperature difference in the afternoon was greater than the light difference in the morning. This was due to the fact that the temperature difference in the afternoon period had a temperature difference caused by the strong light moment and the medium strong light period. The above results were consistent with the findings of Xu Hongjun [6], the low light period did not need to control the temperature and light of the crop, and the temperature of the crop area was within the range of suitable temperature for crop growth.

**Vertical patterns of light and temperature change during periods of medium light intensity.** In the morning time 10:00 ~ 11:00 time period, belonged to the medium light zone 1 section, this time period the shed quilts has been completely opened, the top of the measuring line measuring point temperature value was the highest, the same span height of the higher measuring point light intensity was larger, the measuring point temperature of each measuring line with the time gradually showed an upward trend; in the light intensity of the time period of zone 1, the average outdoor temperature of 8.5 °C, the average light intensity of 79,530 lx; The average temperatures of the measuring points at the top of the same measuring line in lines 1−6 were 31.2°C, 30.9°C, 31.4°C, 32.9°C, 30.7°C, 29.8°C, and the temperature difference between them and measuring point No. 1 of the corresponding measuring line were 4.2°C, 6.6°C, 10°C, 8.5°C, 9.3°C, 7.6°C. The average temperatures at the maximum light measurement points 1−1, 2−5, 3−5, 4−7, 5−6, and 6−5 in the measurement lines 1–6 were 27.0°C, 30.9°C, 30.0°C, 32.9°C, 32.4°C, and 31.1°C; the average temperature differences with the measurement point 1 in each line were 0°C, 6°C, 8.6°C, 8.5°C, 11.0°C, and 8.9°C, respectively; The average light intensity at the maximum light measuring points 1−1, 2−5, 3−5, 4−7, 5−6, 6−5 in the measuring lines 1–6 were: 32047.7 lx, 20653.2 lx, 32550.2 lx, 40991.6 lx, 34635.8 lx, 20219.8 lx, and the difference in the average light intensity at the corresponding measuring point 1 were 0 lx, 5828.2 lx, 27994.5 lx, 35580.0 lx, 29239.6 lx, 16038.9 lx, respectively.

In the afternoon time period from 14:30–15:30, belonging to the medium-light zone 2 section, the top of each measuring line measured the highest temperature value, spanning the same height at the higher measuring point of light intensity was greater, the temperature of the measuring point of each measuring line with the time gradually showed an upward trend; the average outdoor temperature in the medium-light intensity zone 2 time period was 4.5 °C; The average temperature values of the measuring points at the top of the same line in measuring lines 1–6 were: 36.4°C, 40.8°C, 40.2°C, 40.0°C, 41.2°C, 40.1°C, and the temperature difference between them and the measuring point No. 1 of the corresponding line were: 0.7°C, 8.2°C, 9.4°C, 8.5°C, 8.2°C and 7.1°C, respectively. The average temperatures at the maximum light measuring points 1–3, 2–4, 3–6, 4–7, 5–5, and 6–6 in the measuring lines 1–6 were:36.4°C, 38.2°C, 40.2°C, 40.0°C, 39.8°C, and 41.0°C; the average temperature difference with the measuring point 1 in each measuring line were: 0.7°C, 5.6°C, 9.4°C, 8.5°C, 6.8°C, and 8.0°C, respectively; The average light intensity at the maximum light points 1–3, 2–4, 3–6, 4–7, 5–5, 6–6 in the measurement lines 1–6 were: 13638.5 lx, 30027.7 lx, 29393.9 lx, 24368.9 lx, 27597.4 lx, 20029.3 lx, and the difference in the average light intensity at the corresponding point 1 were: −135.4 lx, 23998.9 lx, 24760.4 lx, 20276.7 lx, 22309.9 lx, 15726.8 lx, respectively.

From the above analysis it can be seen that in the middle light period, when the influence of the arch and quilt shading on the top measuring points of measuring line 1 and line 6 of each measuring line was removed, the light intensity was higher and the temperature was higher at the higher measuring points of the same span; The temperature of the mid-light 1 time period was located between 20°C and 30°C, and the maximum light was located between 20,000 lx and 40,000 lx; the temperature of the mid-light 2 time period was located between 30°C and 40°C, and the maximum light was located between 13,000 lx and 30,000 lx. Temperature and light need to be controlled during the mid-light 2 time period, and high temperatures have an inhibitory effect on crop growth and respiration, especially in the crop area, which requires ventilation and cooling treatments.

**Vertical patterns of change in light and temperature during periods of intense illumination.** Strong light time period was located between 11:00–14:30, at this time the light intensity of each measurement point was greater than 25000 lx, the No. 1 measuring line was located in the southernmost part of the crop planting area, was not affected by the crop shading, so it showed the phenomenon of higher light at the bottom; The top measurement points on lines 2–5 were affected by shading from the arch, so the data from the top points were removed, and the top measurement points on line 6 were affected by shading from the arch and the quilts, so the data from points 6–7 and 6–8 were removed.

The light intensity in the greenhouse was positively correlated with the height and the temperature was also positively correlated with the height during the strong light period; the maximum average light in the measuring line 1 ~ 5 was between 34000 lx ~ 44000 lx during this time period, while the maximum average temperature was between 40°C ~ 43°C; the light intensity in the bottom measuring point of the same measuring line was less than 20000 lx, and the temperature in the bottom measuring point was less than 35°C. This environmental condition inhibits the crops in the greenhouse and requires shading and cooling of the greenhouse.

In order to accurately obtain the shading height in the greenhouse, this experiment used k-means to classify the light and temperature data of different measurement points of the same measurement line during the strong light time period, with 2 classes as the standard, so as to define the light and temperature boundary points of different measurement lines, according to the results show that: the temperature boundary point of each measurement line and the light boundary point are located at the position of y = 600 mm or y = 1200 mm. Considering the height of the crop and the internal arching of the greenhouse, a shade controller should be placed at y = 1800 mm. Significance analysis of the light and temperature data at y = 1800 mm and y = 1200 mm showed no difference, so the point at y = 1800 mm can be selected for predictive analysis.

From the above analysis, it can be seen that: due to the regulation of indoor temperature in the high-intensity time period and 2 sections of the mid-afternoon light zone, thus reducing the damage of high temperature to the crop; located in the top of the measurement point of the light intensity was larger, and the light intensity of the bottom of the measurement point of the light intensity was smaller; The temperature of the top measuring point was the highest in the middle light period and the strong light period, and the top measuring point received the heat of the light intensity more than the heat loss between the top measuring point and the outdoor temperature; based on the consideration of the height of the crop growth and the construction of the space facilities, shading measures should be set up at y = 1800mm and ensure that the cooling measures are taken.

#### 3.1.2 Patterns of temperature and light change along the horizontal and vertical directions.

In order to study the change rule of sunlight intensity along the vertical direction of the solar greenhouse, the strong light moment of the sun’s altitude angle is located in the greenhouse facing angle of the smaller moments of analysis, so that you can avoid the arches and crop leaves on the impact of the equipment light, selected 29 January 2025 12:10 moments of each measurement point of the light data and temperature data to analyse.

As the No.1 measuring line was located at the beginning of the crop planting area, it was less shaded by the crop, but the arch was lower from the ground at this position, resulting in the equipment at the upper end of the No.1 measuring line being shaded by the arch; as the No.1 measuring point of each measuring line was the most seriously shaded by the crop, and the shading was not uniform, the measuring point at y = 0 mm of each measuring line was removed; Measurement line 6 was located at the end of the crop planting area, and was also affected by the support frame, the equipment below Y = 1800mm canopy height was more shaded by the crop, and above Y = 1800mm canopy height was more shaded by the greenhouse quilt, so the measurement line 6 was removed for analysis.

As can be seen from Table 4: in different spanning areas at the same height, light intensity gradually increases with increasing *x*. Below the height of the crop canopy at Y = 1800 mm, the maximum temperature difference between the temperature at the smaller *x* position and the temperature at the larger x at the range of *y* = 300 mm, 600 mm, and 1200 mm for the measuring lines 1–5 were: 2.2°C, 3.8°C, and 2.4°C, and the maximum light differences corresponding to them were: −3100 lx, 30.7 lx, −2873.3 lx; Above the height of the Y = 1800 mm crop canopy, the maximum temperature differences between the temperature at the smaller x position and the temperature at the larger x at the range of y = 1800 mm, 2400 mm, and 3000 mm for lines 1–5 were −0.9°C, −0.7°C, and 0°C, with corresponding maximum light differences of −5851.9 lx, −3471.4 lx, and −1271 lx. It can be seen that the light intensity gradually increases with the increase of span at the same height position, both in the crop layer range and outside the crop layer range; in the crop layer range, the temperature of the front part of the solar greenhouse was high, while outside the crop layer range, the temperature of the front part of the solar greenhouse was weak.

**Table 4:** Light and temperature data table for each measurement point. Unit: light (lx),temperature (°C).

Measuring line						
Measuring Point	1	2	3	4	5	6
1	38.1	37.3	32.2	34.3	34.9	36.5
	48433.4	49174.8	36886.6	38604.8	45683.23	43663.4
2	39.0	39.0	38.0	36.8		37.7
	37055.0	39353.2	39863.7	40155.6		43663.4
3	42.1	42.4	39.3	40.5	38.6	38.4
	38179.6	38461.4	38430.7	38830.1	38430.7	36843.5
4		41.2	39.1	40.8	41.5	43.2
		36056.4	38179.8	40697.0	41053.1	35852.0
5		43.3	42.8	43.1	42.3	42.9
		41932.8	42126.1	43821.3	49673.2	31692.1
6			43.5	44.2	44.2	41.9
			38963.2	40970.7	42434.6	41368.4
7				43.1	43.1	43.4
				48347.4	49618.4	29864.4
8						41.1
						18231.6

In the same span with different height areas, with the increase of y, the light intensity was gradually enhanced; in the greenhouse spanning the middle area of Y = 4500 mm and Y = 6000 mm, the No. 2 ~ 5 measuring line in the range of y < 1800mm, the maximum temperature difference between the temperature at the smaller y position and the temperature at the larger y position in the same range of measuring line were: −2.2°C, −1.1°C, −4°C, −2.9°C, and the corresponding maximum light differences were: 3296.6 lx, 1683.9 lx, −541 lx, −2662.4 lx; The maximum temperature difference between the temperature at the smaller x position and the temperature at the larger x in the range y > 1800 mm for lines 3–5 were: −1°C, −1.1°C, −1.9°C, and the corresponding maximum light difference is 3162.9 lx, −7377.4 lx, and −7238.6 lx. It can be seen that within the crop canopy height range, the same span of position increases with height, and the light intensity gradually increases, and the temperature gradually increases, within the 10% error; outside the canopy range, the same span increases with height, and the light intensity gradually increases, and the temperature difference within the canopy range is greater than the temperature difference outside the canopy.

In summary, the same span increases with height, light intensity gradually increases and temperature gradually increases; the same height increases with span, light intensity gradually increases and temperature gradually increases; the temperature varies greatly within the crop canopy, and the temperature and light intensity values at y = 1800 mm were more important.

### 3.2 Change pattern of horizontal CO_2_ concentration

In order to investigate the CO_2_ concentration of the crop at the seedling and flowering stages, the CO_2_ concentration values at y = 600 mm were taken at different spans and analysed.

**Fig 5:**
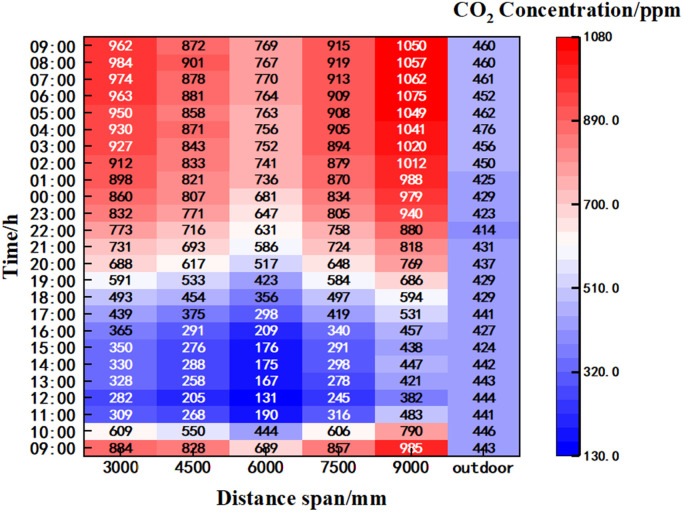
Plot of hourly mean carbon dioxide in greenhouse over time.

At 9:00 the quilts were put away, at 12:20 the upper air vents were opened for ventilation, and at 17:10 the upper air vents were closed and the quilts were put down. As can be seen from Fig 5: the outdoor CO₂ concentration exhibited a consistent and stable level of 440 ppm. During the 9:00–12:20 time interval, the CO₂ concentration value exhibited a decreasing trend within the range of y = 600 mm, x= (3000, 9000) mm,

During the time period 9:00–12:20, the carbon dioxide concentrations at the starting moment of measurement lines 2−6 were 931.4 ppm, 882.7 ppm, 752.0 ppm, 881.5 ppm, and 951.5 ppm, with decreases of 684.8 ppm, 693.8 ppm, 642.7 ppm, 646.7 ppm, and 577.7 ppm. 12:20–13:10, the upwind vent was opened for ventilation, and the carbon dioxide concentrations at each measurement line were 330.9 ppm, 243.3 ppm, 152.9 ppm, 275.4 ppm, and 448.5 ppm, and the increases were 84.3 ppm, 54.4 ppm, 43.8 ppm, 40.6 ppm, and 74.7 ppm. 13:10–15:40 time period, the carbon dioxide concentration of each measurement line was stable, and the carbon dioxide concentration of each line was stable at 334.0 ppm, 278.9 ppm, 172.3 ppm, 290.4 ppm, and 435.6 ppm, with the maximum variation of 39.6 ppm, 49.3 ppm, 28.3 ppm, 27.8 ppm, and 36 ppm. 15: 40–17:10, the carbon dioxide concentration of each measurement line showed an increasing trend, with the increase of 61 ppm, 76.2 ppm, 72.7 ppm, 64.6 ppm, and 81.5 ppm. 17:10–9:00 of the next day, the carbon dioxide concentration of each measurement line showed an increasing trend, with the starting concentration of 412.3 ppm, 363.3 ppm, 262.7 ppm, and 409.6 ppm, 509.0 ppm, and rising at 540.1 ppm, 503.5 ppm, 494.5 ppm, 520.5 ppm, 540.5 ppm.

Line 4 was located in the middle of the crop planting area, the crop photosynthesis absorbed more carbon dioxide, making the concentration value smaller than the concentration value of the other measurement lines. The period between 9:00 and 11:30 saw a gradual increase in light intensity, which in turn led to a corresponding increase in plant photosynthesis and a decrease in carbon dioxide concentration. By midday, the temperature inside the greenhouse had increased to a level that was too high to inhibit crop photosynthesis, and there had been minimal change in the CO_2_ concentration at each measurement line; At the moment of 15:40 the temperature in the indoor gradually decreased, the light intensity decreases leading to a weakening of photosynthesis in the crop, a gradual increase in respiration and a gradual increase in CO_2_ concentration. In general, the CO₂ concentration in the middle area of crop cultivation was low, and the differences in the mean CO₂ concentration of different measurement lines at the same time period during the whole day were relatively small. The changes in CO₂ concentration were closely related to the distribution of crops, photosynthetic intensity and environmental temperature. It was therefore recommended that measures be taken to cool down and replenish the carbon during the time period of 12:00–15:30.

### 3.3 Effect of light on soil temperature change

In order to investigate the change rule of light on soil temperature, the horizontal measurement points were selected in order to analyse the values of light and soil temperature at y = /-150 mm, x = 1500 mm, 3000 mm, 4500 mm, 7 500 mm and 9000 mm; the vertical measurement points were taken at x = 4500 mm, y = 0 mm, −150 mm, −300 mm, −450 mm and −600 mm for analysis.600 mm were analysed.

#### 3.3.1 Effect of light on vertical soil temperature.

**Fig 6:**
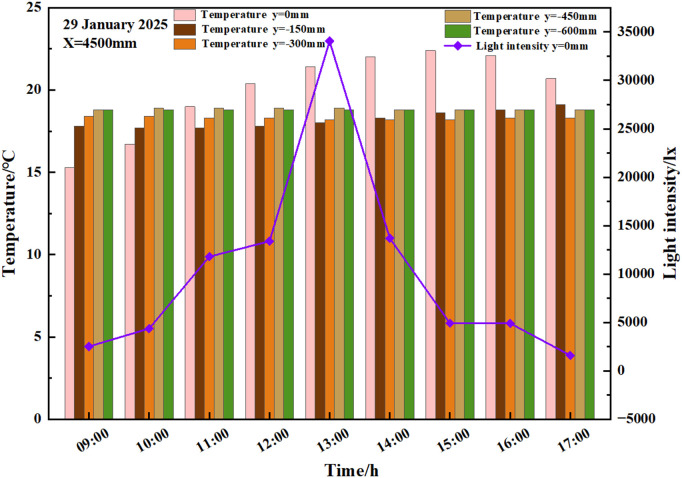
Soil temperature at different depths and soil surface light over time.

From Fig 6: at x = 4500 mm, y = 0 mm, the indoor light intensity from 9:00 opening moment to 12:30 showed an upward trend, 12:30–17:00 moment showed a downward trend. It was observed that the trends in soil temperature at x = 4500 mm, y = 0 mm, −150 mm in the solar greenhouse were consistent with the trends in light. While the soil temperatures at y = −300 mm, y = −450 mm, and y = −600 mm were maintained at approximately 18.3°C, 18.8°C, and 18.8°C, respectively, with the maximum temperature difference not exceeding 0.1°C on that day. Consequently, the analysis of soil temperatures was required at depths of 0 mm and −150 mm, espectively.

The soil temperature of y = 0 mm and y = −150 mm increased from 15.3°C and 17.8°C to 22.3°C and 18.5°C, respectively, during the time period from 9:00–14:30 (the cut-off time of strong light); soil surface light increased from 1761 lx to 3481.2 lx during the time period of low light from 9:00–10:00, and the soil temperature of y = 0 mm and y = −150 mm increased by 1.4°C and −0.1°C, respectively; 10:00 ~ 11:00 medium-light time period soil surface light increased from 3481.2 lx to 27382.4 lx, and soil temperature of y = 0 mm and y = −150 mm increased by 2.3°C and 0°C, respectively; 11:00 ~ 14:30 strong-light time period soil surface light gradually increased from 27382.4 lx to 54133.4 lx and then gradually decreased to 17750 lx, the soil temperature of y = 0 mm and y = −150 mm increased by 3.2°C and 0.7°C, respectively; the soil surface light of the medium light time period from 14:30–15:30 decreased from 17750 lx to 12259.2 lx, and the soil temperature of y = 0 mm and y = −150 mm increased by 0.2°C and 0.1°C; soil surface light decreased from 12259.2 lx to 1104.8 lx in the low light time period from 15:30–17:00, the soil temperature at y = 0 mm showed a decreasing trend, while the soil temperature at y = −150 mm showed an increasing trend, and the soil temperature difference between the corresponding time periods of y = 0 mm, and y = −150 mm were −1.6°C and 0.4°C, respectively.

As demonstrated above, there was a significant impact of light on the change in soil temperature in the greenhouse with y = 0 mm. In contrast, the change in soil temperature in the greenhouse with y = −150 mm was less influenced by light. The trend of soil temperature change in the greenhouse with −150mm < y < −300 mm was unclear, and the soil temperature in the greenhouse with y < −300 mm remained stable.

#### 3.3.2 Effect of light on soil horizontal temperature.

**Fig 7:**
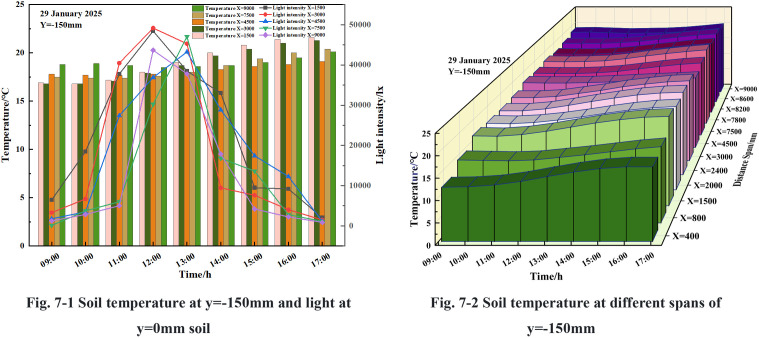
Variations in soil temperature and light at y = −150 mm.

From Figs 7-1 and 7-2, it can be seen that the intensity of light in the solar greenhouse exhibited an upward trend from 9:00–12:30, coinciding with the opening of the greenhouse, and a downward trend from 12:30–17:00, corresponding to the closing of the greenhouse. The vertical distance from the soil surface in the greenhouse was consistent, and the soil temperature in the range of 1500mm < x < 9000mm exhibited a downward trend9:00–12:00, followed by an upward trend at 12:00–17:00; the soil temperature trends in the solar greenhouse at y = −150 mm, x = 400 mm, 800 mm, 1500 mm, 3000 mm showed an upward trend throughout the day, and the soil temperature greenhouse in the range of x < 800mm was at the lowest temperature. Throughout the day, the soil temperature trend in the solar greenhouse at y = −150 mm, x = 4500 mm, 7500 mm, 9000 mm exhibited a stable trend prior to 12:30, subsequently displaying an upward trend. The maximum temperature difference between the measurement points was less than 0.5°C.

The light intensity at various points within the solar greenhouse was measured over a period of 6 hours, from 12:30–17:00. The measurements were taken at specific points, namely y = −150 mm, *x* = 1500 mm, 3000 mm, 4500 mm, 7500 mm, and 9000 mm. The recorded light intensity decreased from 51826.7 lx, 53300.3 lx, 541 The light intensity decreased from 51826.7 lx, 53300.3 lx, 5413.3 lx, 57354.6 lx, 43663.3 lx, respectively, to 2013.9 lx, 3349.3 lx, 1104.7 lx, 1110.4 lx, and 872 lx. Correspondingly, the soil temperatures in the solar greenhouse at y = −150 mm, x = 400 mm, 800 mm, 2000 mm, 2400 mm, 1500 mm, 3000 mm, increased by 2.8°C, 2.9°C, 3.1°C, 3.0°C, respectively, 3.2°C, 3.1°C; soil temperatures at y = −150 mm, x = 4500 mm, 7500 mm, 7800 mm, 8200 mm, 8600 mm, 9000 mm, increased by 1.2°C, 2.6°C, 2.9°C, 2.7°C, 2.6°C, 1.6°C, respectively, in the solar greenhouse.

From the above, it can be deduced that the soil temperature of different spans of the same depth in the solar greenhouse was affected by the light intensity, with the greatest warming evident at 12:30, when the greenhouse reached its maximum light intensity. The greenhouse at *x* = 4500 mm was found to be a significant demarcation point, with the soil temperature changes in the x < 4500 mm range showing greater variation, and those in the x > 4500 mm range exhibiting less variation.

### 3.4 Effect of light on wall temperature change

In order to investigate the change rule of light on the vertical temperature and lateral temperature of the wall, the light data of different measurement points of the No.7 measurement line should be taken and the light data at the corresponding height should be linearly inserted for analysis; the height of the wall should be taken as y = 0 mm, 800 mm, 1600 mm, 2400 mm and 3200 mm at the cross-section. The distance from the inner surface of the wall along the above cross-section is then taken as 0 mm, 200 mm, 400 mm, 600 mm and 800 mm from the inner surface of the wall.

#### 3.4.1 Changing law of light on wall temperature along the lateral direction.

**Fig 8:**
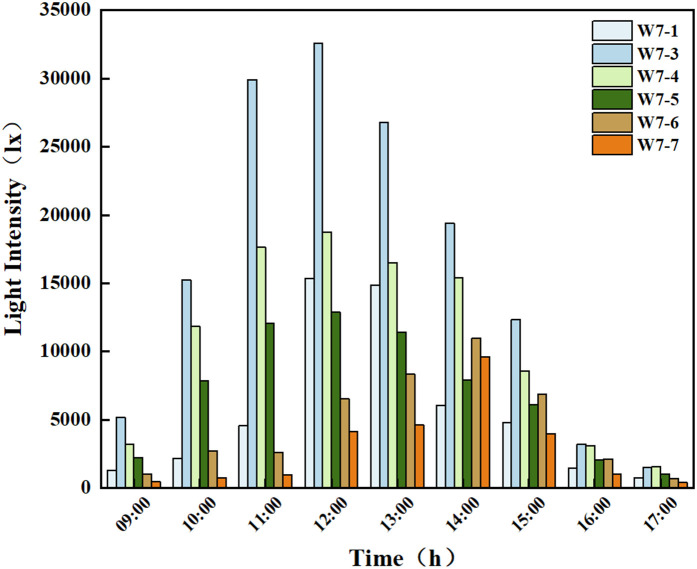
Wall lighting.

**Fig 9:**
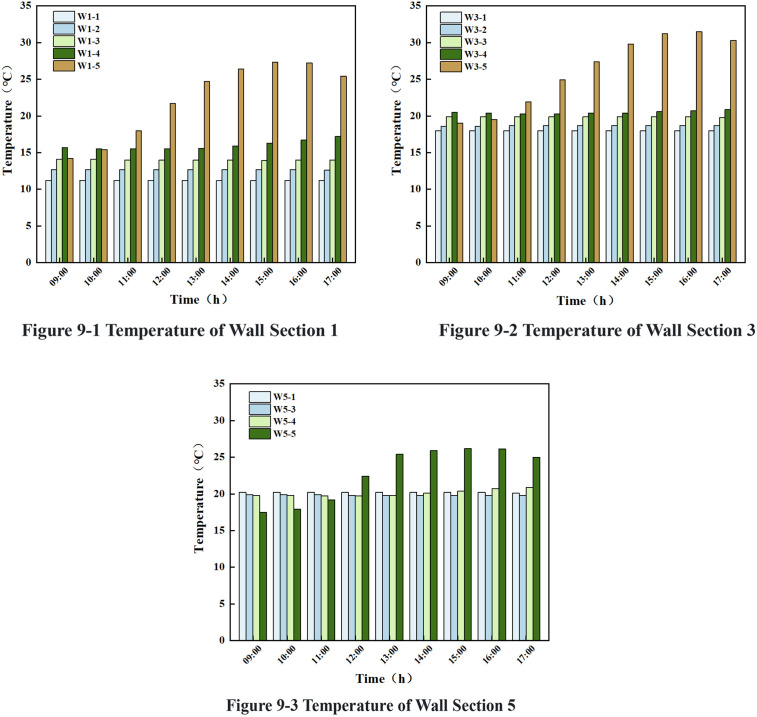
Lateral wall temperature.

The temperature of the wall at varying depths from the inner surface of the wall was analysed by taking the heights of y = 0 mm, 1600 mm and 3200 mm, as illustrated in Figs 8 and 9 below. In the time period from 9:00–17:00, the temperature of the wall at the same height from the inner surface of the wall at a horizontal distance of (400,800) mm range does not exceed 0.1 °C, and there was a trend of change in the temperature in (0,200) mm.

From Fig 8 and Fig 9, it can be seen that: In the time period from 9:00–17:00, in the W1, W3, W5 cross-section, the change of solar light intensity has no effect on the temperature at the same height and the horizontal distance from the inner surface of the wall of 400 mm, 600 mm, 800 mm, and the temperature change of these measurement points with time was less than 0.2°C;The temperature decreases with increasing horizontal distance from the inner surface of the wall at the same height, except for the Y = 0 mm (W5 section) measurement point.

Within the W1 section, the initial temperatures at the horizontal distances of 400 mm, 600 mm, and 800 mm from the inner surface of the wall were 14.1°C, 12.7°C, and 11.2°C, respectively. Within the W3 section, the initial temperatures at the horizontal distances of 400 mm, 600 mm, and 800 mm from the inner surface of the wall were 19.9°C, 18.6°C, and 18.0°C, respectively. Within the W5 section, the initial temperatures at the horizontal distance from the inner surface of the wall at 400 mm, 800 mm were 19.9°C, 20.2°C.

Within the W1 section, the W1-4 temperature decreased gradually with time during the initial period from 9:00–11:30, with an initial temperature of 15.7°C and a decrease of 0.3°C; during the subsequent period from 11:30–17:00, the increase was 1.8°C. Conversely, the W1-5 temperature exhibited an upward trend during the initial time period, from 9:00–15:30, with an initial temperature of 14.2°C and an increase of 13.3°C. However, during the subsequent time period, from 15:30–17:00, a downward trend of 2°C was observed. During the interval from 9:00–10:00, the temperature difference between W1-4 and W1-5 exhibited a gradual decrease, with a maximum difference in temperature of 1.9°C and a minimum difference of 0.1°C. Conversely, from 10:00–17:00, the temperature difference between W1-5 and W1-4 exhibited a marked increase, with a maximum difference of 11°C and a minimum difference of 0.3°C.

Within the W5 segment, the W5-4 temperature remained within the range of 18.7°C and 18.8°C during the 9:00–12:00 interval. During the 12:00–17:00 interval, the W5-4 temperature exhibited a gradual increase, with an initial temperature of 19.8°C and an increase of 1.1°C. The W5-5 temperature exhibited a gradual increase with time during the 9:00–15:30 time period, with an initial temperature of 17.5°C and an increase of 8.9°C. Conversely, the W5-5 temperature exhibited a gradual decrease from 15:30–17:00, with an initial temperature of 26.4°C and a decline of 1.4°C. During the time period from 9:00–11:10, the temperature difference between W5-4 and W5-5 gradually decreased, with a maximum temperature difference of 2.3°C and a minimum temperature difference of 0°C. Conversely, from 11:10–15:30, the temperature difference between W5-4 and W5-5 exhibited a marked increase, with a maximum temperature difference of −5.9°C and a minimum temperature difference of −0.3°C. During the 15:30–17:00 time period, the temperature difference between W5-4 and W5-5 began to gradually decrease again, with a maximum temperature difference of −5.6°C and a minimum temperature difference of −4.1°C.

From the above analysis, it can be seen that: the significant difference of light on the wall temperature was mainly located on the surface of the wall, and the influence on the temperature of the measuring point at the distance of 200 mm from the inner surface of the wall at different heights is very small, with a maximum of 1.5°C. Additionally, during the whole day time period, there was no influence on the temperature of the measuring point at the horizontal distance of 400 mm, 600 mm and 800 mm from the inner surface of the wall.

#### 3.4.2 Changing law of light on the wall temperature along the vertical direction.

**Fig 10:**
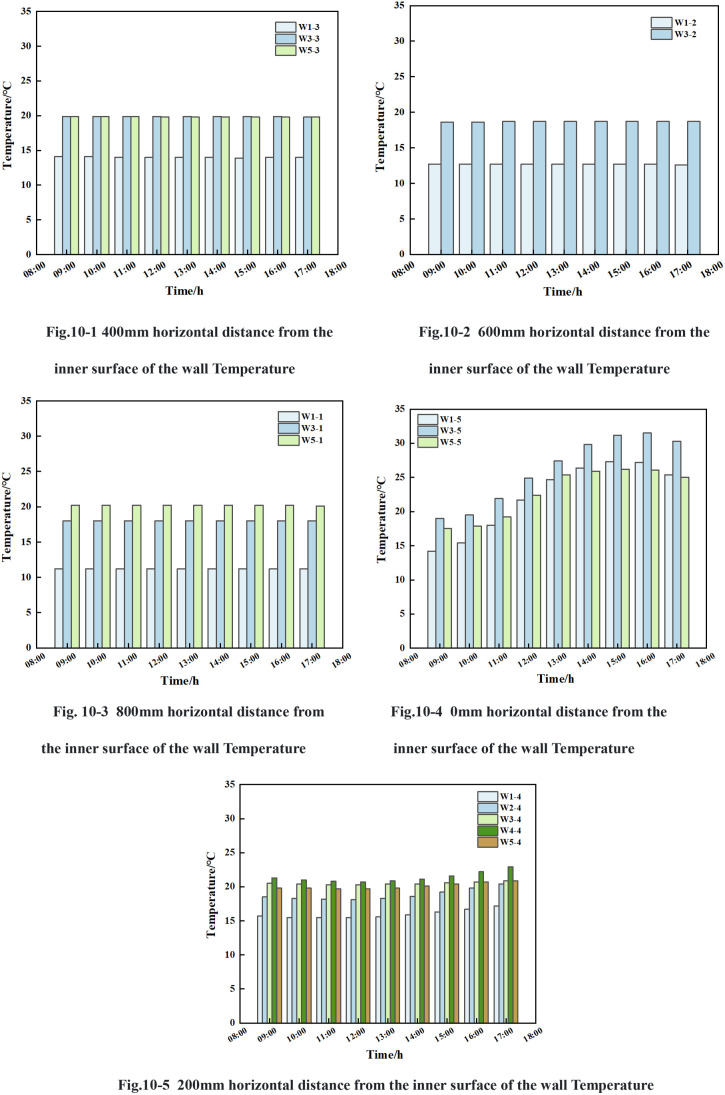
Vertical temperature of the wall.

From Fig 10, it can be seen that: in the time period of 9:00 ~ 17:00, the temperature values of the wall at the horizontal distance from the inner surface of the wall of 400 mm, 600 mm and 800 mm in the cross section of W1, W3 and W5 were less than 0.2°C, and the temperatures at the same horizontal distance from the inner surface of the wall increase with the increase of the height. The light variations of W1, W2, W3, W4, and W5 cross sections were 395.7 lx ~ 27105.6 lx, 631.1 lx ~ 13294.8 lx, 1208.45 lx ~ 17669.75 lx, 1524.6 lx ~ 31758.2 lx, and 643.5 lx ~ 31529.0 lx, respectively.

As illustrated in Fig 10, the trend of the wall temperature values at a horizontal distance of 0 mm from the inner surface of the wall within the cross-sections of W1, W3, and W5 exhibited a clear pattern over the specified time period from 9:00–17:00. The following temperature increases were observed: From 9:00–15:30, the temperatures of W1-5, W3-5, and W5-5 exhibited a gradual increase, with initial temperatures of 14.2°C, 19.0°C, and 17.5°C, respectively. The increases were 13.3°C, 12.4°C, and 8.9°C, respectively. Conversely, from 15:30–17:00, a gradual decline in temperature was observed, with initial temperatures of 25.5°C, 28.4°C, and 25.4°C, respectively, indicating a drop of 2°C, 1.2°C, and 1.2°C.Among them, the temperature of W3 section was the highest, in the time period of 9:00 ~ 11:30, the temperature of W5-5 was higher than the temperature of W1-5, and the temperature difference between the two measurement points decreased gradually with the change of time, and the maximal temperature difference between W5-5 and W1-5 was 3.3°C, and the minimal temperature difference was 0.1°C.In the time period of 11:30 ~ 17:00, the temperature of W1-5 was higher than the temperature of W5-5, and their maximum temperature difference was 1.2°C and the minimum temperature difference was 0.3°C.

The temperature values of the wall, located at a horizontal distance of 200 mm from the inner surface of the wall within the W1-W5 section, exhibited a specific trend during the designated time period from 9:00–17:00. The temperatures of W1-4, W2-4, and W3-4, W4-4, and W5-4 decreased slowly, with the initial temperatures of 15.7°C, 18.5°C, 20.5°C, 21.3°C, and 19.8°C, with decreasing ranges of 0.2°C, 0.4°C, 0.2°C, 0.6°C, and 0.1°C, respectively. During the time period from 12:00–17:00, the temperatures of W1-4, W2-4, W3-4, W4-4, and W5-4 gradually increased, with increasing ranges of 1.7°C, 2.3°C, 0.6°C, 2.2°C, and 1.2°C, respectively. The temperature of the W4 section was higher than that of the W5 cross-section, and the temperature difference between W4-4 and W5-4 decreased from 1.5°C to 1.1°C during the time from 9:00–11:00. 11:00–14:30, the temperature difference between W4-4 and W5-4 was maintained between 1°C and 1.1°C. And during the time period from 14:30–17:00, the temperature difference between W4-4 and W5-4 increased from 1.1°C to 2°C. The maximum temperature difference between W4-4 and W5-4 was 2°C, and the minimum temperature difference was 1°C.

In summary, the change of light intensity did not cause a temperature change at a horizontal distance of 400 mm, 600 mm and 800 mm from the inner surface of the wall; however, it had a greater effect on the temperature at a horizontal distance of 0 mm from the inner surface of the wall, and a smaller effect on the temperature at a distance of 200 mm from the inner surface of the wall. The W3 and W4 sections exhibited higher temperatures, while the W1, W2, and W5 sections demonstrated lower temperatures.

### 3.5 LSTM prediction model

In combination with the preceding analyses, the light difference was at Y = 1800 at the light difference demarcation point and canopy height, and X = 4500 mm at the soil demarcation point. The light impact on the wall was primarily within a horizontal distance of 200 mm from the inner surface of the wall, and the temperature increase was significant during the period of high-intensity light. It is recommended that high temperature suppression in the latter half of the crop be reduced during the 11:00–15:00 time period, and that reflecting the light from this region to the wall be used to increase the wall’s heat storage during the day. A deep learning model was constructed for the measurement point data at X = 4500, Y = 1800, with the aim of providing data support for the control of high temperature hours.

**Fig 11:**
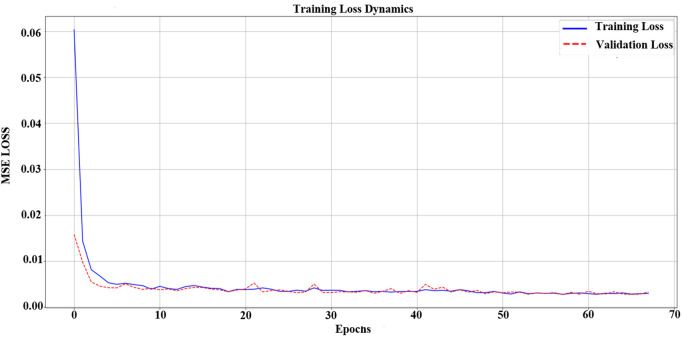
Loss map of training set and validation set.

To enhance the prediction of the greenhouse environment under various weather conditions, light and temperature data from January 11, 2025, to January 16, 2025 (cloudy and hazy), and from January 31, 2025, to February 5, 2025 (sunny), were selected for training an LSTM prediction model (Fig 11, Table 5).

**Table 5:** MAE and R^2^ values under different weather conditions.

evaluation indicators		cloudy and hazy	sunny
temperature	MAE	0.46	0.62
	R^2^	0.99	0.99
light	MAE	1753.98	1615.54
	R^2^	0.94	0.95

As shown in Table 5, the model’s prediction of temperature under cloudy and hazy conditions exhibited a low mean absolute error (MAE) of 0.46°C, whereas under sunny conditions, the MAE value increased slightly to 0.62°C. For the prediction of light intensity, the MAE value was 1753.98 lx under cloudy and hazy conditions, while it was 1615.54 lx under sunny conditions. In terms of the coefficient of determination (R²), the R² value for temperature prediction was as high as 0.99 under both cloudy and hazy conditions and sunny conditions, indicating that the model predicts temperature changes with very high accuracy and explanatory power. For light intensity, the R² value was 0.94 under cloudy and hazy conditions and 0.95 under sunny conditions, indicating that the model effectively captured the trend of light intensity changes under different lighting conditions. Therefore, in practical application, a large number of data sets can be trained to predict high light intensity conditions in the greenhouse using the LSTM model for early warning, enabling growers to take timely measures based on the light and temperature conditions in the greenhouse.

#### 3.5.1 Analysis of LSTM prediction results.

**Fig 12:**
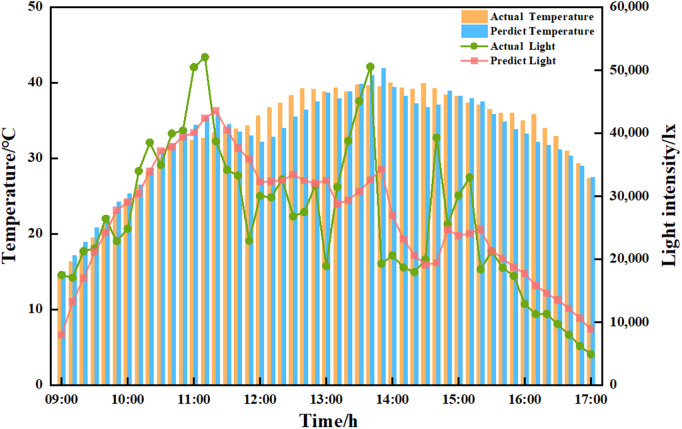
Comparison of predicted data and test data results.

Temperature prediction was conducted at the measuring point, which spanned a distance of 4500 mm and a height of 1800 mm for the solar greenhouse. The test values from 9:00 on 5 February 2025–17:00 on 5 February 2025 were selected for comparison with the predicted values of the LSTM prediction model. The results are presented in Fig 12.

As can be seen from Fig 12: the discrepancy between the predicted and measured temperatures was minimal, with a maximum disparity of 3.8°C. The prediction result was deemed to be satisfactory. A substantial discrepancy was observed between the predicted and measured light levels. This discrepancy can be attributed to the influence of factors such as sunlight in the rotation, the angle of the equipment, and the presence of arch frame shading. These factors contributed to variations in the light levels at specific moments in time, resulting in significant differences between the predicted and actual light levels. However, during the majority of the time, the actual light levels and the predicted light difference values remained within the range of 10% or less, which was consistent with the predictions.

#### 3.5.2 Residual analysis.

**Fig 13:**
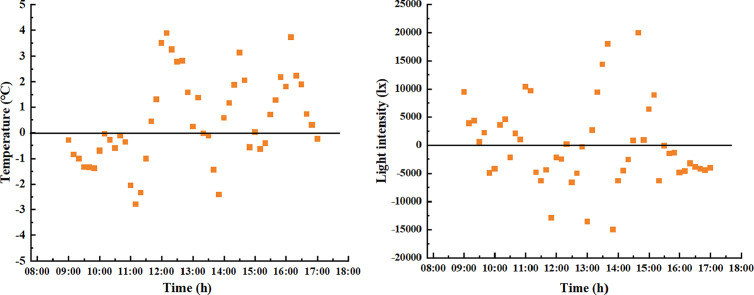
Residual plots for temperature and light intensity.

To more accurately verify the accuracy of the LSTM prediction model, a residual analysis was conducted by comparing the experimental values from 9:00 AM to 5:00 PM on February 5, 2025, with the predicted values from the LSTM model. The residual plots for temperature and light intensity were shown in the figures below (Fig 13):

In summary, the results of temperature prediction and light prediction were satisfactory, so the LSTM model can be applied to predict the future temperature and light conditions by shading the back half of the greenhouse cross-section in advance and reflecting it back to the wall, thus improving the growth of crops in the north part of the greenhouse and increasing the heat storage in the wall.

## 4. Conclusion

This paper explores the effect of light distribution in a greenhouse on the temperature of different parts of the greenhouse through experimental measurements and deep learning models, and its conclusions were as follows:

[1] The difference between indoor and outdoor light levels was substantial. For the specific solar greenhouse in Shanxi, the maximum light absorption indoors was 75% of the outdoor light, and the intensity of sunlight absorbed by the wall was 50% of the outdoor light.
[2] As shown in Table 6, the indoor light was differentiated along the vertical and longitudinal directions of the greenhouse, and the indoor light variance along the vertical direction was greater than that along the horizontal direction. The light variance along the vertical direction was greater in the range of light intensity within the range of Y=(0,1800) mm. The light intensity variance along the horizontal direction was greater within the range of x = [0, 4500) mm, and smaller within the range of x = [4500, 9000] mm.
[3] The variability of indoor light on soil temperature along the lateral direction was found to be significant, with a more pronounced effect on temperature along the x=[400,4500) mm range and a comparatively smaller effect on temperature in the x=[4500,90 0] mm range; the interval of variability of indoor light on soil temperature along the vertical direction was located in the [0,150] mm range, and there was no effect on temperature in the [300,600] mm range.
[4] The variability of indoor lighting on wall temperature along the horizontal direction exhibited a greater range of variation in the [0,200] mm horizontal distance from the inner surface of the wall, and no discernible effect was observed in the range of [400,800] mm. In addition, the variability of indoor lighting on wall temperature along the vertical direction was found to be larger in the range of [800,1600] mm height distance from the ground, and had a smaller effect in the [0,800) mm and [1600,3200] mm.
[5] The LSTM model demonstrated a high degree of accuracy in its predictions. It is imperative that the greenhouse temperature and light levels are meticulously regulated during the 11:00–15:00 time period.

**Table 6:** Influence of Light on the Temperature Range within the Greenhouse.

	Significant Influence	Minimal Influence
The Impact of Solar Radiation on Air Temperature	X=[0, 4500) mm	X = [4500, 9000]mm
	Y= (0,1800) mm	Y= (1800,3200]mm
The Impact of Solar Radiation on Soil Temperature	X =[400,4500) mm	X = [4500, 9000]mm
	Y= [0, −150]mm	Y= [−300, −600]mm
The Impact of Solar Radiation on Wall Temperature	X = [0,200]mm	X = [400,800]
	Y= [800,1600]mm	Y=[0,800) mm and [1600,3200]mm

Note: The horizontal distance x for the impact of solar radiation on wall temperature refers to the distance from the inner surface of the wall. All other measurements are consistent with the established coordinate system.

## 5. Future prospects

Although this study has made progress on the existing foundation, it still faces some limitations and deficiencies. Firstly, although the experiment primarily focused on temperature and light, the growth environment of crops is also influenced by multiple factors such as humidity and carbon dioxide concentration. Future research will incorporate a broader range of environmental variables for systematic study to comprehensively assess the interactive effects of these factors on crop growth. Secondly, the LSTM prediction method used in this study has its limitations. Although it can effectively capture the long-term dependencies in time series data, it is highly sensitive to input variables and has a high computational cost. Combining machine learning methods with other prediction techniques, such as physical models and hybrid models, will be investigated in the future to enhance prediction accuracy and computational efficiency.

## Supporting information

S1 Data The data in paper analysis. (XLSX)
